# Brain diffusion tensor imaging in dogs with degenerative myelopathy

**DOI:** 10.1111/jvim.16248

**Published:** 2021-08-19

**Authors:** Melissa J. Lewis, Jeremy L. Shomper, Baye G. Williamson, Daniella P. Vansteenkiste, Katherine F. Bibi, Stefanie H. Y. Lim, Joseph B. Kowal, Joan R. Coates

**Affiliations:** ^1^ Department of Veterinary Clinical Sciences College of Veterinary Medicine, Purdue University West Lafayette IN USA; ^2^ Department of Veterinary Medicine and Surgery University of Missouri, College of Veterinary Medicine Columbia MO USA

**Keywords:** dog, imaging biomarker, SOD1, spinal cord, white matter

## Abstract

**Background:**

Degenerative myelopathy (DM) in dogs shares similarities with superoxide dismutase 1‐associated human amyotrophic lateral sclerosis (ALS). Brain microstructural lesions are quantified using diffusion tensor imaging (DTI) in ALS patients.

**Objective:**

Characterize brain neurodegenerative changes in DM‐affected dogs using DTI.

**Animals:**

Sixteen DM‐affected and 8 control dogs.

**Methods:**

Prospective observational study. Brain DTI was performed at baseline and every 3 months on DM‐affected dogs and compared to controls. Fractional anisotropy, mean diffusivity, axial diffusivity, and radial diffusivity were calculated on specified regions of interest. Gait scores (0, normal to 14, tetraplegia) were assigned at each scan. Diffusion tensor imaging values in DM‐affected dogs were compared to controls, gait scores, and evaluated over time.

**Results:**

Mean age was 5.7 years (SD 3.2) in controls and 9.7 years (SD 1.4) in DM‐affected dogs. In DM‐affected dogs, mean baseline gait score was 4 (SD 1), and mean score change from baseline to last scan was 4.82 (SD 2.67). Nine dogs had ≤3 scans; 7 had >3 scans. Accounting for age, no differences in DTI indices were identified for any brain or proximal spinal cord regions between DM‐affected dogs and controls (*P* > .05). Diffusion tensor imaging values poorly correlated with gait scores (*R* ^2^ < .2). No significant changes were identified in diffusion indices over time (*P* > .05).

**Conclusions and Clinical Importance:**

Diffusion tensor imaging indices did not differentiate DM‐affected from control dogs, detect longitudinal changes, or differentiate disease severity. Findings do not yet support brain DTI as an imaging biomarker.

**Abbreviations**
AD: axial diffusivity
ALS: amyotrophic lateral sclerosis
DM: degenerative myelopathy
DTI: diffusion tensor imaging
FA: fractional anisotropy
MD: mean diffusivity
MRI: magnetic resonance imaging
RD: radial diffusivity
ROI: regions of interest
SOD1: superoxide dismutase 1

## 1 INTRODUCTION

Degenerative myelopathy (DM) is a late adult‐onset, progressive neurodegenerative condition in dogs that shares similarities with some forms of superoxide dismutase 1 (SOD1)‐associated human amyotrophic lateral sclerosis (ALS).^1^, ^2^, ^3^ Degenerative myelopathy causes widespread degeneration of sensory and motor neuronal pathways of both the central and peripheral nervous systems. Definitive diagnosis is attained with histopathology postmortem but presumptive diagnosis is achieved by confirming the *SOD1* genotype coupled with compatible clinical signs and advanced imaging to rule out other causes of thoracolumbar myelopathy.^1^

Objective disease biomarkers capable of enhancing diagnosis and tracking disease progression are needed to assist in the development of therapeutic interventions for both dogs with DM and people with ALS. Various blood, cerebrospinal fluid, and electrodiagnostic biomarkers are described in DM‐affected dogs and people with ALS.^4^, ^5^, ^6^, ^7^, ^8^, ^9^ Advanced imaging techniques have also been investigated as potential noninvasive imaging biomarkers in people with ALS with a growing number of explorations in dogs with DM.^4^, ^6^, ^10^, ^11^, ^12^ Diffusion tensor imaging (DTI) is a variation of diffusion‐weighted magnetic resonance imaging (MRI) that leverages the cellular motion of water to quantify microstructural lesions, primarily in white matter.^13^, ^14^ In the brains of ALS patients, DTI demonstrates widespread white matter changes compared to healthy controls,^4^, ^12^, ^15^, ^16^, ^17^, ^18^, ^19^, ^20^, ^21^, ^22^, ^23^ and longitudinal changes are detectable in some brain areas.^15^, ^16^, ^20^, ^24^, ^25^, ^26^ Corticospinal tract changes are variably correlated with disease progression, validated clinical scales used in ALS patients and electrodiagnostic tests.^16^, ^18^, ^21^, ^22^, ^23^, ^24^, ^25^

Spinal cord DTI in DM‐affected dogs identifies alterations in diffusivity parameters in regions with more severe lesion burden compared to controls but is less able to differentiate mildly affected areas.^11^ Brain DTI is not reported in DM but there can be histologic changes in the brains and proximal spinal cords of DM‐affected dogs. While 2 case series did not identify overt degeneration in the brain,^27^, ^28^ there are variable lesions in the red nucleus, vestibular nuclei, dentate nucleus, nucleus gracilis, and fasciculus gracilis in the cervical spinal cord.^29^, ^30^ Brain DTI also detects microstructural changes in other neurodegenerative diseases in dogs.^31^, ^32^ This suggests that it is a feasible modality to evaluate brain neurodegenerative changes in dogs with DM. Diffusion tensor imaging of the spinal cord faces several challenges in part because of the small size of the spinal cord contributing to a low signal to noise ratio.^33^, ^34^ We speculate that brain DTI might be able to detect lesions in DM‐affected dogs complementing what has been reported for spinal cord DTI and provide longitudinal data in these dogs.

The objectives for this project were to use DTI to characterize the location and extent of degenerative changes in the brain of DM‐affected dogs compared to healthy controls, and to determine the relationship between DTI indices, clinical disease severity, and disease progression in DM‐affected dogs. We hypothesized that DTI would detect degenerative changes in the brain of DM‐affected dogs relative to healthy control dogs primarily affecting the white matter regions as well as gray matter areas previously reported to be abnormal histologically in DM‐affected dogs. We further hypothesized such changes would correlate with clinical severity and be able to detect disease progression over time.

## 2 MATERIALS AND METHODS

### 2.1 Study dogs

Healthy control and DM‐affected dogs were prospectively recruited as part of various clinical trials being performed at the University of Missouri Veterinary Health Center. Healthy control dogs were included if they were systemically healthy with no history of neurologic disease, had a neurologic examination that did not reveal abnormalities at the time of imaging, and had a known *SOD1* genotype. Degenerative myelopathy‐affected dogs were included if they were homozygous for the E40K SOD1 mutation, demonstrated clinical signs compatible with DM and had a thoracolumbar MRI with standard sequences excluding other causes and supporting the presumptive diagnosis of DM. Degenerative myelopathy‐affected dogs were participants in the following clinical trials: SOD1 epitope dimeric interface (SEDI) immunotherapy, canine SOD‐1 antisense oligonucleotide (ASO) trial, and an AAV.iSOD1 gene therapy trial with trial‐specific exclusion criteria. No treatment interventions were demonstrated to impact disease progression in any of the aforementioned clinical trials.

### 2.2 Clinical evaluation

Signalment and duration of neurologic signs (defined as the interval between the onset of gait deficits as described by the owner and initial MRI) were recorded for each dog. Disease stage and gait scores were assigned for each dog at each study visit. Disease stage was determined using published criteria where stage 1 = upper motor neuron paraparesis and general proprioceptive ataxia, stage 2 = nonambulatory paraparesis or paraplegia, stage 3 = tetraparesis, and stage 4 = flaccid tetraplegia, widespread muscle atrophy and bulbar dysfunction.^1^ Gait was quantified using an adapted open field scale ranging from 0 to 14 where 0 refers to a normal gait and 14 to tetraplegia.^5^, ^35^ Scores greater than 7 correspond to being nonambulatory.

### 2.3 Imaging acquisition

Brain MRI with DTI were performed on a 3.0‐T Toshiba/Canon Titan scanner (Canon Medical Systems USA, Tustin, California) using Canon XL‐knee coil and orthopedic multichannel (6) speed coil with transmit and receive capabilities. Using the same protocol, imaging was acquired once in control dogs and at baseline and approximately every 3 months in DM‐affected dogs for a variable total number of scans per dog. All dogs were positioned in sternal recumbency and the following standard sequences were acquired in the transverse and sagittal plane: T1 weighted pre and postcontrast, T2 weighted, fluid‐attenuated inversion recovery, proton density, and gradient echo/T2*. Imaging parameters for the conventional MRI are outlined in the supporting information, Table S1. Brain MRIs were reviewed by a boarded neurologist to ensure there were no structural abnormalities. Diffusion tensor imaging was acquired in the transverse plane using the following imaging protocol: slice thickness of 3.0 mm with 0.3 mm gap, voxel size of 1.56 × 1.56 × 3.0 mm, 30 directions, field of view of 20 × 20 cm, *b*‐value of 1000 s/mm^2^, TR: 5630 ms, and TE: 90 ms. Scan time was approximately 6:12 minutes. While the spinal cord was not specifically imaged, all scans continued caudal to the foramen magnum and included the proximal portion of the cervical spinal cord.

### 2.4 Image processing

Raw diffusion DICOM images were converted to 4D NIfTI format using MRIcron with diffusion direction files (.bvec, .bval) generated by reformatting the PreSet_30axis.tbl file provided by the manufacturer (Canon Medical Systems USA). Using FSL commands, data were corrected for eddy current and motion distortion and an automated mask was used to remove extraneural tissues. A diffusion tensor model was fitted to processed images using the FSL “dtifit” command which provides a matrix‐valued tensor for each voxel.

Using T2 weighted transverse images for reference, regions of interest (ROI) were manually created by 1 author for all scans for specific gray and white matter brain regions using the processed images. We chose to focus on regions where lesions might be present, extrapolated from DTI studies in ALS patients^18^, ^19^, ^21^ (eg, body of the corpus callosum, internal capsule) and based on histologic abnormalities that occur in the brains and proximal spinal cord of dogs with DM (eg, red nucleus, dorsal columns).^29^, ^30^ Control regions that we anticipated would be unaffected were not specifically targeted. The following areas were initially considered for inclusion: corpus callosum, internal capsule, corona radiata, pre and postcruciate gyrus, caudate nucleus, thalamus, brainstem, red nucleus, caudal cerebellar peduncle, cerebellum (including the deep cerebellar nuclei), proximal spinal cord, dorsal column of proximal spinal cord. Image distortions or artifacts precluded consistently identifying the pre and postcruciate gyrus on all scans and was removed from consideration. Corona radiata, caudate nucleus, thalamus, red nucleus, and brainstem regions were eliminated because of challenges identifying the borders of these regions and consistently applying ROI across dogs and between scans within a given dog. Regions of interest for the body of the corpus callosum, internal capsule, caudal cerebellar peduncle, cerebellum, proximal spinal cord, and dorsal column of the proximal spinal cord were included in the analysis. The size and shape of each ROI was variable but conformed to the structural edges of the specific brain region for the body of the corpus callosum, caudal cerebellar peduncle, and spinal cord (circumferentially and for the dorsal columns). For the internal capsule and cerebellum, the ROI were located within the structure of interest, focusing on creating a region in the same area and of similar size across scans. For all ROI, care was taken to avoid including extra‐axial structures when constructing ROI and to be as consistent as possible between dogs and scans. Fractional anisotropy (FA), mean diffusivity (MD), axial diffusivity (AD), and radial diffusivity (RD) were calculated for each ROI providing quantitative information on the magnitude and direction of diffusivity.

### 2.5 Statistical analysis

Summary statistics for healthy and DM‐affected dogs were reported as mean (SD) or median (range), as appropriate. Normality was determined using the Shapiro‐Wilk test. Mean age between the 2 groups was compared using a student's *t* test. Regression analysis and an ANCOVA incorporating age as a covariate were used to compared mean DTI values for each ROI between DM‐affected and control dogs. Among DM‐affected dogs, DTI values were compared to gait scores and evaluated over time using Pearson correlation and univariate split‐plot approach, respectively. For all analyses, *P* < .05 was considered significant. The Holm‐Bonferroni correction method was used to account for multiple comparisons with *P*_*a*_ used to denote adjusted *P* values.

## 3 RESULTS

### 3.1 Study dogs

Eight healthy control dogs were included with a mean age of 5.7 years (SD 3.2). This group included 5 Boxers (5 females, 1 male), 2 Chesapeake Bay Retrievers (2 females), and 1 Labrador Retriever (male). E40K SOD1 genotype was homozygous normal in 3 dogs, heterozygous in 2, and homozygous affected in 3 dogs. None of the control dogs went on to develop signs compatible with DM over a period of 2.5 to more than 5 years of follow‐up. Sixteen DM‐affected dogs were enrolled including 8 Boxers (2 females, 4 males), 4 Pembroke Welsh Corgis (1 female, 1 male), 2 Chesapeake Bay Retrievers (2 males), 1 German Shepherd Dog (1 female), and 1 Anatolian Shepherd. Mean age at enrollment was 9.7 years (SD 1.4). Degenerative myelopathy‐affected dogs were significantly older than healthy control dogs (*P* = .001).

### 3.2 DTI in DM‐affected compared to control dogs

Fifty‐two total scans were performed in DM‐affected dogs including 9 dogs with ≤3 scans and 7 dogs with >3 scans (Table 1). Four or more scans were associated with at least 9 months of follow‐up after baseline. Mean FA, MD, AD, and RD values for each ROI are listed in Table 2. Accounting for the age difference between groups, significant differences were not identified in any of the DTI indices between DM‐affected and control dogs (*P* > .05, all comparisons).

**TABLE 1:** Distribution of the 52 total diffusion tensor imaging (DTI) scans performed among the 16 degenerative myelopathy (DM)‐affected dogs

Number of serial DTI scans performed per dog	Number of dogs
1	5
2	2
3	2
4	2
5	3
6	1
8	1

**TABLE 2:** Mean FA, MD, AD, and RD values for each ROI in DM‐affected compared to healthy control dogs

ROI	Mean (SD) FA		*P* value	Mean (SD) MD (×10^−3^ mm^2^/s)		*P* value	Mean (SD) AD (×10^−3^ mm^2^/s)		*P* value	Mean (SD) RD (×10^−3^ mm^2^/s)		*P* value
	DM	Control		DM	Control		DM	Control		DM	Control	
CC	0.61 (0.09)	0.68 (0.07)	.29	0.58 (0.06)	0.58 (0.06)	.99	1.02 (0.08)	1.09 (0.13)	.59	0.36 (0.07)	0.31 (0.06)	.43
IC	0.64 (0.06)	0.72 (0.07)	.51	0.64 (0.04)	0.63 (0.06)	.61	1.19 (0.11)	1.27 (0.09)	.36	0.36 (0.05)	0.31 (0.07)	.85
Cd Ped	0.45 (0.1)	0.44 (0.08)	.29	0.70 (0.09)	0.68 (0.08)	.95	1.07 (0.13)	1.02 (0.08)	.30	0.51 (0.10)	0.52 (0.10)	.55
CBM	0.31 (0.06)	0.35 (0.08)	.88	0.70 (0.04)	0.67 (0.05)	.46	0.95 (0.07)	0.93 (0.06)	.50	0.58 (0.04)	0.55 (0.07)	.64
SC	0.43 (0.06)	0.57 (0.08)	.16	0.72 (0.07)	0.72 (0.1)	.86	1.07 (0.11)	1.20 (0.15)	.67	0.55 (0.05)	0.47 (0.13)	.28
DC	0.40 (0.09)	0.54 (0.08)	.28	0.88 (0.02)	0.74 (0.1)	.62	1.23 (0.30)	1.21 (0.18)	.82	0.69 (0.16)	0.51 (0.13)	.44

*Note*: *P* < .05 denotes significance.

Abbreviations: AD, axial diffusivity; CBM, cerebellum; CC, corpus callosum; Cd Ped, caudal cerebellar peduncle; DC, dorsal column of proximal cervical spinal cord; DM, degenerative myelopathy; FA, fractional anisotropy; IC, internal capsule; MD, mean diffusivity; RD, radial diffusivity; ROI, regions of interest; SC, proximal cervical spinal cord.

### 3.3 DTI and disease stage

Mean duration of neurologic abnormalities (from onset of abnormal signs to initial imaging) was 5.7 months (SD 3.3). All dogs were in disease stage 1 at enrollment. Among the 11 dogs with more than 1 scan, 5 dogs remained in stage 1 throughout, 3 dogs progressed from stage 1 to stage 2, and 3 dogs progressed from stage 1 to stage 3 during the study. Mean baseline gait score was 4 (SD 1), consistent with ambulatory paraparesis. Among the 11 dogs with more than 1 scan, mean change in gait score between baseline and last scan was 4.8 (SD 2.7), representing a progression to nonambulatory paraparesis. Among the 7 dogs with >3 scans, mean baseline gait score was 3.6 (SD 1.5) and mean change from baseline was 6.2 (SD 2.1). No imaging was performed in any dogs with stage 4 (end stage) disease.

**FIGURE 1:**
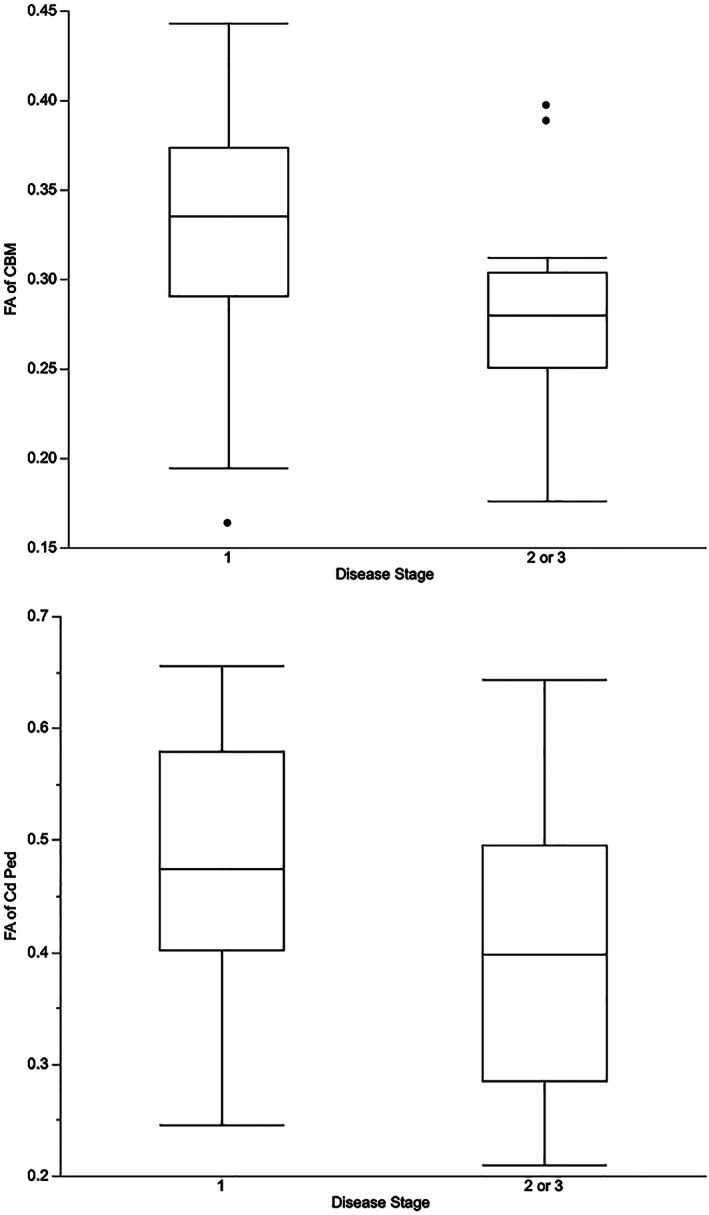
Relationship between FA and disease stage for the cerebellum (*P* = .02, *P*_*a*_ = .1) and caudal cerebellar peduncle (*P* = .04, *P*_*a*_ = .2). CBM, cerebellum; Cd Ped, caudal cerebellar peduncle; FA, fractional anisotropy

Among the DM‐affected dogs, DTI values correlated poorly with gait scores (*R* ^2^ < .2, all comparisons). There were too few dogs with advanced disease (3 with stage 3 and none with stage 4 disease at the time of last MRI) to investigate associations between individual disease stages and DTI values. When dogs were categorized as disease stage 1 versus stage 2 or 3, FA of the cerebellum and caudal cerebellar peduncle were each lower in dogs in stage 2 or 3 (*P* = .02, .04, respectively) though the differences were no longer significant after adjusting for multiple comparisons (*P*_*a*_ = .1, *P*_*a*_ = .2, respectively) (Figure 1). No other relationships between DTI indices and disease stage were identified.

### 3.4 DTI and disease progression

**FIGURE 2:**
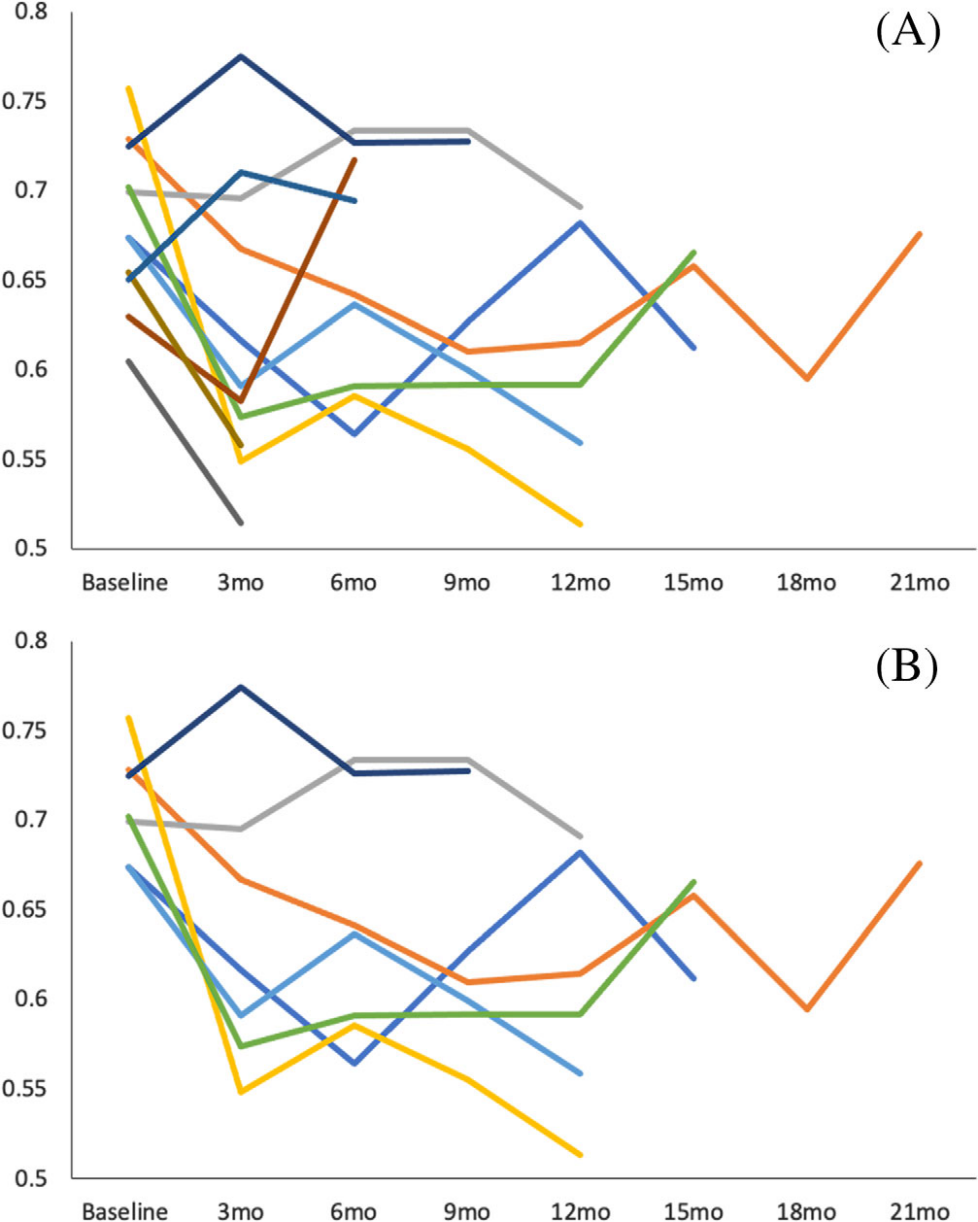
Change in FA of the IC over time in (A) 11 DM‐affected dogs with >1 scan (*P* = .07) and (B) 7 DM‐affected dogs with >3 scans (*P* = .03, *P*_*a*_ = .17). DM, degenerative myelopathy; FA, fractional anisotropy; IC, internal capsule; mo, months

For the 11 DM‐affected dogs with >1 scan, there was variability in DTI values between individuals and changes in quantitative values over time within individuals were small. In these dogs, FA of the internal capsule decreased over time but the difference was not significant (*P* = .07) (Figure 2A). When considering just the 7 dogs with >3 scans (representing a timeframe of at least 9 months between baseline and last scan), there was a decrease in FA of the IC over time (*P* = .03). However, the difference was no longer significant once adjusted for multiple comparisons (*P*_*a*_ = .17) and interindividual variability remained high (Figure 2B). No longitudinal changes were identified for MD, AD, or RD for any brain regions (*P* > .05, all comparisons).

## 4 DISCUSSION

DTI of the brain and proximal spinal cord was unable to differentiate DM‐affected dogs from healthy controls after accounting for differences in age between the groups. Among DM‐affected dogs, these DTI values did not demonstrate a clear relationship with disease progression or clinical disease severity as measured by gait scores. In general, DTI of the brain and proximal spinal cord was influenced by age, there was high interindividual variability and longitudinal changes in DTI values were small over the course of this study.

This study was not able to identify structural differences in brain and proximal spinal cord regions of DM‐affected dogs relative to healthy controls using DTI. While FA in multiple areas was generally lower and MD and RD of the dorsal column were generally higher compared to controls, these differences were not significant. In people with ALS, brain DTI can detect widespread, predominantly white matter degenerative changes compared to healthy controls, even early in the disease course.^4^, ^12^, ^15^, ^16^, ^17^, ^18^, ^19^, ^20^, ^21^, ^22^, ^23^ However, there is variability in which brain areas are reported to be abnormal using DTI, there is notable overlap with values in controls, and postmortem studies confirm unpredictable distribution of lesions in the brain of ALS patients.^12^, ^15^, ^17^, ^36^, ^37^ Despite these inconsistencies, DTI is suggested to be potentially useful as a diagnostic biomarker in ALS.^12^, ^15^, ^18^ In dogs with DM, there are no other studies of brain DTI and there are relatively few reports detailing brain neurodegeneration.^27^, ^28^, ^29^, ^30^ These studies describe either a lack of overt degeneration in the brain^27^, ^28^ or lesions variably present in the red nucleus, vestibular nuclei, dentate nucleus, and nucleus gracilis.^29^, ^30^ While this suggests that brain lesions are possible in dogs with DM, questions remain regarding the extent and timing of development of brain lesions. It is possible the brain and proximal spinal cord lack substantial abnormalities or they only develop in end‐stage disease compared to the thoracolumbar spinal cord, where the lesion burden is prominent and histologically well‐characterized.^1^, ^27^, ^38^ In the only study of spinal cord DTI in DM, FA is significantly lower in severe DM cases compared to control dogs, but only within areas with more severe lesion burden (caudal cervical, mid‐thoracic, and cranial lumbar spinal cord).^11^ Consistent with our findings, no differences in FA or MD are reported for ROI in the cranial cervical spinal cord in that study.^11^ Additionally, FA is not different between more mildly affected dogs and healthy control dogs other than in the cranial lumbar spinal cord, and overlap between groups is large.^11^ Using brain DTI (including the proximal spinal cord) as a diagnostic biomarker might, therefore, not be useful in dogs with DM due to lack of notable brain neurodegeneration or, if present, limited observed changes in the setting of early‐stage disease.

The majority of our healthy control dogs were heterozygous or homozygous affected by the SOD1 mutation. Studies in asymptomatic SOD1 mutation carriers in people report conflicting results,^39^, ^40^ but abnormalities in DTI indices consistent with neurodegeneration in the internal capsule are described in people with no clinical signs of ALS.^39^ Additionally, in patients diagnosed with ALS, DTI detects microstructural abnormalities in brain regions that remain clinically silent (ie, for which there are no clinical signs of dysfunction).^15^ It is possible that some of the dogs in our control group were presymptomatic with neurodegeneration already occurring within their brains. However, regardless of genotype, none of the control dogs went on to develop clinical signs of DM over a period of multiple years and histopathology was not available for this study. Further imaging studies using healthy control dogs that do not carry any copies of the SOD1 mutation and thorough histopathologic evaluation of the brain and proximal spinal cord in DM‐affected and clinically asymptomatic carrier dogs are warranted. Additionally, studies in people with ALS combining structural (eg, imaging‐based) and functional (eg, electrophysiologic‐based) modalities can more comprehensively evaluate the integrity of cortical motor neurons.^40^ Broad characterization of the neurodegenerative changes associated with the SOD1 mutation in dogs with and without clinical disease will help to determine if DTI of the brain can be a useful diagnostic biomarker in dogs with DM.

In the current study, FA of the internal capsule decreased over time but the change was no longer significant once corrected for multiple comparisons. We had only 7 dogs that were followed for at least 9 months and marked clinical progression during the study period was uncommon. There was also high interindividual variability and the longitudinal changes in DTI values were small within a given dog. These factors might have impacted our ability to detect changes over time. Furthermore, there are no imaging‐based, longitudinal studies in DM in dogs to which to compare our results. In people with ALS, there is variability between studies regarding associations between DTI and disease progression. In several longitudinal studies, FA decreases, especially in the internal capsule, and more extensive regions of the brain become affected over time.^15^, ^16^, ^20^, ^24^, ^25^, ^26^ However, changes over time are inconsistent when considering group (pooled) versus individual patient data and are typically only significant when considering scans performed many months (>8 months) from baseline.^15^, ^16^, ^26^ It is also noted that changes in gray matter regions, in particular cerebellar gray matter, show greater progression over time compared to white matter.^15^ These studies suggest that ALS patients must be monitored over a relatively long timeframe for DTI evidence of progression to become apparent and that progression varies between individuals, the type of brain tissue, and the specific brain region. The current study focused primarily on white matter regions, but it is possible progressive neurodegeneration would have been more apparent in gray matter regions as has been reported by people.^15^ As noted above, the lack of detectable longitudinal changes might also reflect the differences in lesion burden between ALS patients and DM‐affected dogs. It is possible that brain lesions in dogs are not substantial or progressive enough to be detected via DTI.

The cerebellum and caudal cerebellar peduncles were abnormal in dogs with more advanced disease (stage 2 or 3) compared to stage 1. However, these changes were not significant after correcting for multiple comparisons, no dogs were imaged with end‐stage (stage 4) disease, and DTI values correlated poorly with gait scores. While FA values of the caudal cervical and thoracolumbar spinal cord have shown moderate negative correlations with gait scores, correlations are weak for FA values of the cranial cervical spinal cord.^11^ FA values of the cranial cervical spinal also show no differences between control, mild, and severely affected groups.^11^ Similar to DTI of the spinal cord, our results suggest that brain and proximal spinal cord DTI might be less useful in the setting of milder disease or in central nervous system regions with less prominent neurodegenerative changes. The connection between DTI and disease severity is also inconsistent in ALS patients. Several studies failed to identify a relationship between DTI indices and standard clinical measures such as the revised ALS functional rating scale, respiratory function testing, and disease duration.^18^, ^21^, ^23^ Others report that FA and MD do correlate with the revised ALS functional rating scale,^16^, ^20^, ^22^, ^24^ though DTI indices are not better at detecting change than that simple clinical scale.^20^ While the role of DTI as a marker of disease severity remains unclear, the differences noted in the cerebellum between dogs of different disease stages might support further evaluation of this brain region.

Limitations of this study include the small sample size and that healthy control dogs were not uniform in their SOD1 status nor age or breed matched to our DM‐affected dogs. While we accounted for the age difference in our statistical model, future studies utilizing DTI should compare dogs with DM to age and ideally breed matched controls with further consideration given to the influence of SOD1 genotype. Utilizing a larger number of dogs that are followed over a consistently longer period of time might also help to overcome the large interindividual variability and enhance the ability to detect small but significant differences between DM‐affected and control dogs and longitudinal changes over time in DM‐affected dogs. However, more prolonged studies would also require accounting for the influence of aging on longitudinal changes in DTI indices. Neurodegenerative changes are well‐established in the brains of human ALS patients,^2^, ^4^ but descriptions of the extent and progression of brain lesions in dogs with DM are limited.^27^, ^28^, ^29^, ^30^ More extensive histologic examination of the brains of DM‐affected dogs is warranted in dogs at different severities of progression and regardless, spinal cord imaging might prove more relevant in DM. We also used an ROI‐based approach to calculate DTI indices, but another analysis method such as tract‐based spatial statistics might be more useful, especially when analyzing multiple scans performed over time. It is also possible that combining DTI with other MRI techniques (eg, functional MRI, magnetic resonance spectroscopy, etc.) or functional modalities (eg, transcranial magnetic stimulation) might enhance the sensitivity to identify structural and functional changes DM‐affected dogs as has been described in people with ALS.^17^, ^40^

Overall, our preliminary results showed that DTI failed to identify overt neurodegenerative changes in the brain and proximal spinal cord of dogs with DM, perhaps due to minimal or absent neurodegeneration in these regions. While brain DTI might have some utility in disease progression or more advanced disease stages, findings do not currently support this modality as a noninvasive, imaging biomarker in dogs with DM.

## CONFLICT OF INTEREST DECLARATION

Authors declare no conflict of interest.

## OFF‐LABEL ANTIMICROBIAL DECLARATION

Authors declare no off‐label use of antimicrobials.

## INSTITUTIONAL ANIMAL CARE AND USE COMMITTEE (IACUC) OR OTHER APPROVAL DECLARATION

Approved by the University of Missouri IACUC (protocol numbers 7349 and 9242).

## HUMAN ETHICS APPROVAL DECLARATION

Authors declare human ethics approval was not needed for this study.

## Supporting information

**Table S1** Imaging protocol for conventional MRI scans.
