# Supplementary material for: Brain diffusion tensor imaging in dogs with degenerative myelopathy
Source: J Vet Intern Med. 2021 Aug 19;35(5):2342–9. doi: 10.1111/jvim.16248 (PMC8478048; doi:10.1111/jvim.16248)
Supplement: Supplementary file 1 — Table S1 Imaging protocol for conventional MRI scans. [file JVIM-35-2342-s001.pdf]

Supporting information

S1. Imaging protocol for conventional MRI scans.

Sequence	TE	TR	Slice Thickness (mm)	FOV (cm)	Gap (mm)
T1 PRE	10	770	3	18 x 18	.3
T1 POST	10	770	3	18 x 18	.3
T2 Axial	120	6837	3	18 x 18	.3
FLAIR	120	10000	3	18 x 18	.3
PD	20	2484	3	18 x 18	.3
T2*	6.8	500	4	18 x 18	.3
